# Osseous Choriostoma of the Upper Lip

**DOI:** 10.22038/ijorl.2020.41909.2387

**Published:** 2020-07

**Authors:** Ashwin-Chandra Veni, Kannan Ashokan, Krithika-Chndrasekar Sekar, Parimala D, Kanmani-Shanmuga Sundaram, Yesoda Aniyan

**Affiliations:** 1 *Department of Oral Medicine and Radiology, SRM Dental College, Chennai, Tamil Nadu, India.*; 2 *Department of Oral Medicine and Radiology, Chettinad Dental College, Chennai, Tamil Nadu.*

**Keywords:** Choriostoma, Mature bone, Osseous choriostoma, Osteocytes

## Abstract

**Introduction:**

Choristoma is a non-neoplastic growth of normal tissues in non-indigenous site of origin. Osseous Choristoma is an ectopic bone formation which is a rare entity with 72 cases has been reported in the tongue, 15 cases in the buccal mucosa, 1 case in the lower lip.

**Case Report:**

A 43 year old male patient experienced a mild, intermittent, dull aching type in the upper lip. An excisional biopsy was done by placing a single incision under local anesthesia. To our knowledge case is first to be reported in the upper lip as a result of reactive pathogenesis.

**Conclusion:**

As per literature, surgical excision is the treatment of choice for osseous Choristoma. Malignant transformation has not been reported yet.

## Introduction

Choristoma is a non-neoplastic growth of normal tissues in non-indigenous site of origin. The term Choristoma was first coined by krolls et al. Choristoma does not posses the characteristic feature of a neoplasm. Choristoma is common among the age group of 2^nd^ to 6^th^ decade. It can be formed from various tissues like bone, cartilage, muscle, glial cells, salivary glands, sebaceous glands. They are named after the tissue of origin (Eg: Osseous Choristoma, Cartilaginous Choristoma). The tongue is the most predominant sites affected intraorally followed by buccal mucosa, palate (1,2).

There are two types of theories involved in the formation of choristoma. 

a) Developmental theory. 

b) Reactive or Post- traumatic theory.

Developmental theory states that the entrapment of undifferentiated mesenchymal cells proliferates into a sessile or a pedunculated mass. According to Adhikari et al, the derivatives of brachial arches are trapped to proliferate within the normal cells in alien site (3). The entrapment of undifferentiated mesenchymal cells in the facial regions is from the embryonic brachial arches I, II and III. The future development of an osseous lesion in the soft tissue remains to be an appealing theory of its origin. Another theory stated in the form of Choristoma is a reactive or posttraumatic theory. As the name suggests, reactive formation of tissues in non-indigenous location secondary to site of trauma (4).

## Case Report

A 43 year old male patient came to the department of Oral medicine and Radiology with the chief complaint of pain in the upper lip for the past 15 days. The patient experienced a mild, intermittent, dull aching type in the same region**. **His Past medical history was non-contributory and in the past dental history the patient revealed the history of trauma from road traffic accident 15 years ago, during which the patient had suffered from lip laceration and fracture of anterior teeth. Laceration in the lip was sutured immediately and later root canal treatment and crown placement was done. He further added that, 2 years after the trauma, he had developed pain and swelling in the upper lip in relation to 11, 12. A minor surgical procedure was done and a tooth spicule was removed. Constitutional signs and review of all systems appears to be normal on general examination. On local examination, inspection revealed no abnormality in the upper lip, labial mucosa and the entire oral cavity. On palpation a painful tiny hard mass measuring about 5 mm approx was palpable in the upper lip 0.5 cm lateral to the midline in relation to 11 and 12. On hard tissue examination prosthetic crown was present in relation to 11, 12. Considering the chief complaint, history and correlating with the clinical findings the mass in the upper lip was provisionally diagnosed as a foreign body in the upper lip. 

**Fig 1:**
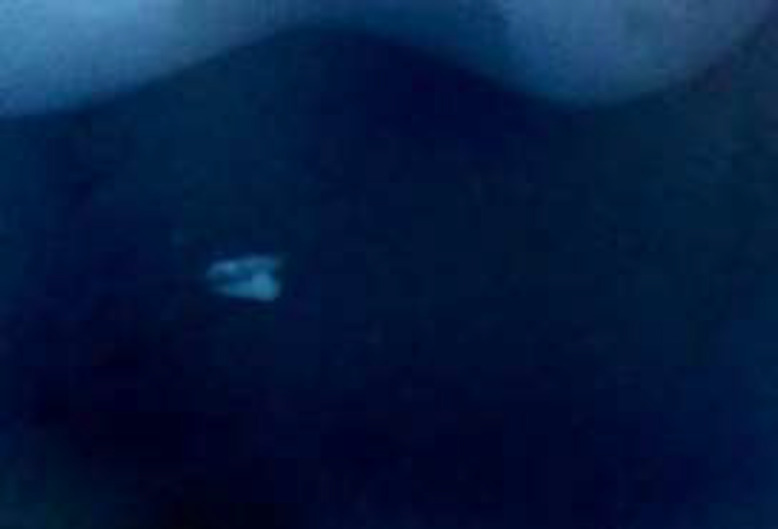
Intraoral periapical radiograph with soft tissue exposure to the upper lip

An intra oral Periapical radiograph of the upper lip revealed a single radiopaque mass measuring about 3 X 2 mm in size (Fig .1).

**Fig 2:**
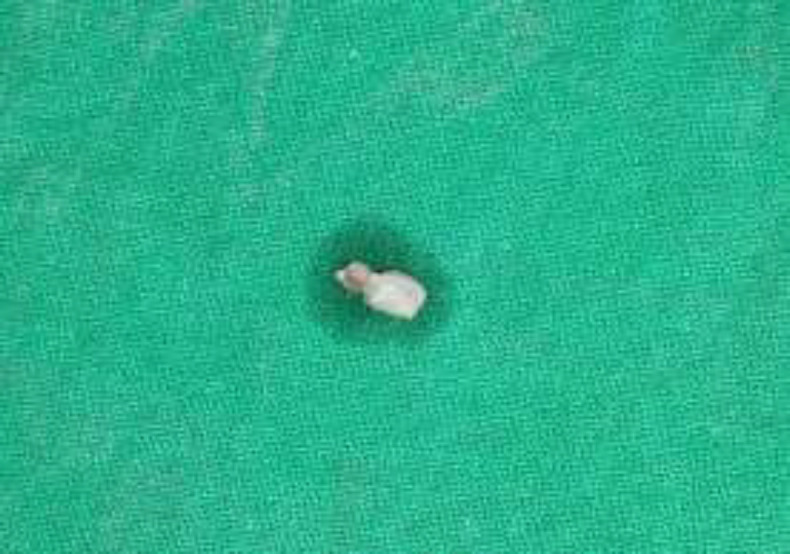
The macroscopic view of the excised tissue through a single incision placed from the labial mucosa.

Blood investigation was performed which revealed to be within normal limits. Then an excisional biopsy was done by placing a single incision under local anesthesia (Fig.2). 

**Fig 3:**
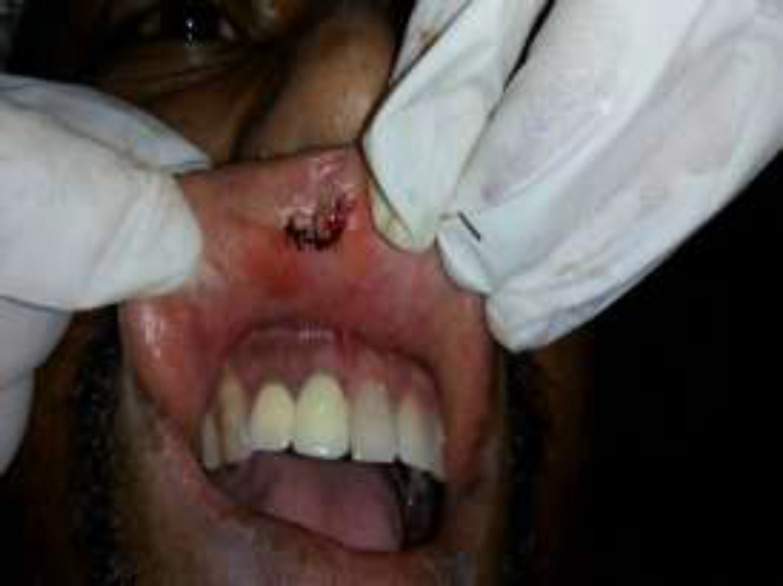
This image shows sutures placed to close the incision line.

All procedures were performed under aseptic condition (Fig.3). 

A review was done after 7^th^day, first month, third month and after 6 month. All necessary check radiographs were taken. The wound healing was satisfactory.

**Fig 4:**
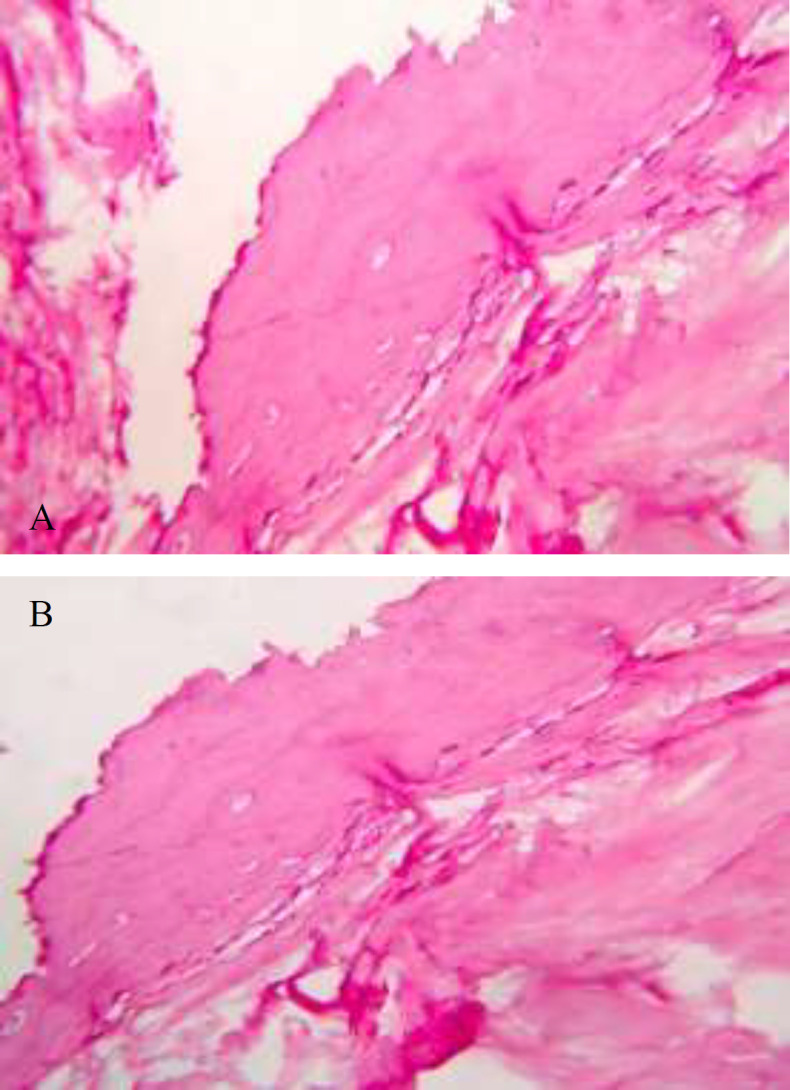
**A-** Histopathological examination shows connective tissue mass with interspersing trabeculae of matured bone and osteocytes in lacunae surrounded by loose to densely arranged collagen fibers. **B-** Image showing osteocytes and loose connective tissue.

Histopathological examination shows the connective tissue mass with interspersing trabeculae of mature bone and osteocytes in lacunae surrounded by loose to densely arranged collagen fibers (Fig.4 (a,b)). Hence the final diagnosis was suggestive of osseous Choristoma. 

## Discussion

Intra oral Choristoma are rare. As per literature there are only 72 cases in tongue mostly situated near the foramen ceacum around the circumvallated papilla, 15 cases in the buccal mucosa, one in palate and one in labial mucosa. T.s. Bastian et al, described a case of Choristoma of the labial mucosa and the etiology was given as unknown. Here we unfold a different presentation of Choristoma in the upper lip. The most probable etiology in this case could be a reactive lesion due to trauma (5). 

Intra oral osseous Choristoma is histologically a clear mass of lamellar bone with the haversian canals around a tight fibrous connective tissue covered with stratified epithelium. Choristoma has to be differentiated from that of a hamartoma, which is a focal overgrowth of haphazardly arranged cells at the normal site of origin. Teratoma is another condition where all three germ layers are mostly involved and presented in non-indigenous location (6). Osteoma is benign progressively enlarging true neoplasm of bone originating from osteogenic tissue and is always associated with skeletal structure. In the present case the biopsy was done without the epithelium hence the differential diagnosis of fibro epithelial polyp with metastatic bone growth couldn’t be ruled out. At any point the mass cannot be considered as a foreign body or a tooth remnant owing to trauma because of the presence of viable mature bone at the site of trauma fulfilling the criteria of post traumatic or reactive theory of Choristoma.

The history of trauma led us to give a diagnosis of foreign body of the upper lip initially. The histological report is characteristic to that of osseous Choristoma. Another reason for excluding the diagnosis of foreign body is the presence of trauma history before 15 years and the absence of multinucleated giant cell histologically. 

Surgical excision is the treatment of choice for osseous Choristoma. Malignant transformation has not been reported yet. There is no evidence of recurrence is mentioned in the literature (7).

## Conclusion

This case report presents a case of osseous choriostoma involving the upper labial mucosa. Surgical removal is the treatment of choice based on site, size and accessibility. Recurrence is uncommon.
